# Leukemia cutis with concomitant acrodermatitis chronica atrophicans and chronic B‐cell lymphocytic leukemia: complete clearance under doxycycline therapy

**DOI:** 10.1111/ddg.15868

**Published:** 2025-08-30

**Authors:** Sven‐Niklas Burmann, Thilo Gambichler, Jürgen C. Becker, Frank Oellig, Alexander Kreuter, Ulrich Peter Wehry

**Affiliations:** ^1^ Department of Dermatology Venereology and Allergology Helios St. Elisabeth Hospital Oberhausen University Witten/Herdecke Oberhausen Germany; ^2^ Department of Dermatology Christian Hospital Unna Unna Germany; ^3^ Department of Dermatology Dortmund Hospital gGmbH University Witten/Herdecke Dortmund Germany; ^4^ Translational Skin Cancer Research DKTK Partner Site Essen/Düsseldorf West German Cancer Center Department of Dermatology University Duisburg‐Essen and German Cancer Research Center (DKFZ) Heidelberg Germany; ^5^ Institute of Pathology Mülheim an der Ruhr Mülheim an der Ruhr Germany

**Keywords:** Acrodermatitis chronica atrophicans, borreliosis, chronic B‐cell lymphocytic leukemia, doxycycline, leukemia cutis, lymphoproliferative diseases

Dear Editors,

Leukemia cutis (LC) is a rare cutaneous manifestation in hematologic malignancies. It arises from secondary infiltration of leukemic cells into the skin, although the pathomechanism is not yet fully understood.^1^, ^2^, ^3^, ^4^ In this case report, we describe a patient who was diagnosed with both LC as a manifestation of chronic lymphocytic leukemia (CLL) and acrodermatitis chronica atrophicans (ACA). The underlying CLL was identified only during dermatopathological analysis. Following oral doxycycline treatment for Lyme disease, complete regression of the LC was observed.

A 67‐year‐old man presented with asymptomatic, disseminated subcutaneous nodules that had been present for 6 months, progressively increasing in both number and size (Figure 1a). Additionally, around the same time, large, cushion‐like, livid plaques developed on the forearms and hands (Figure 1b). No relevant internal medical history was known, and the patient was not on any medication. Histopathological examination of a subcutaneous nodule from the trunk revealed superficial and deeper perivascular lymphocytic infiltration without plasma cells or epidermal involvement. Immunohistochemical expression of CD5, CD20, and CD23 suggested cutaneous infiltration of a B‐cell lymphoma, indicative of LC (Figure 2a–d). A subsequent punch biopsy of a livid plaque on the forearm revealed CD138‐positive plasma cells. Due to the clinical and histological appearance of ACA on the forearms and hands, PCR analysis was performed on both this biopsy and the LC, and both tested positive for *Borrelia (B.) burgdorferi* DNA. Furthermore, a positive IgG immunoblot (antigens: DbpA, Osp17, OspC, p21, p30, p39, p43, p84, and VlsE) and a positive Lyme disease serology (*B. burgdorferi* IgG/IgM EIA) confirmed the diagnosis of chronic Borrelia infection. Additional diagnostics revealed leukocytosis (96,000/µl; normal range: 4,400–11,000/µl) with markedly elevated B‐cell counts (53,320/µl; normal range: 100–500/µl). A subsequent bone marrow biopsy revealed infiltration by a low‐grade non‐Hodgkin lymphoma consistent with CLL. Mutation analysis revealed intermediate genetic risk (unmutated IGHV status, no del(17p) or TP53 mutation, no complex karyotype). Imaging revealed diffuse lymphadenopathy and splenomegaly.

**FIGURE 1:**
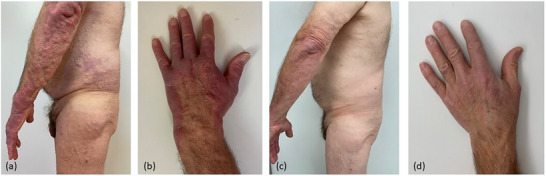
(a) Disseminated subcutaneous nodules are predominantly visible in the trunk and thigh. Additionally, large, partly succulent, livid plaques are visible on the forearms. (b) Cushion‐like infiltrated, livid plaques on the hands. (c) Complete healing of all skin changes on the trunk. (d) Complete healing of all skin changes on the limbs following oral doxycycline treatment.

**FIGURE 2:**
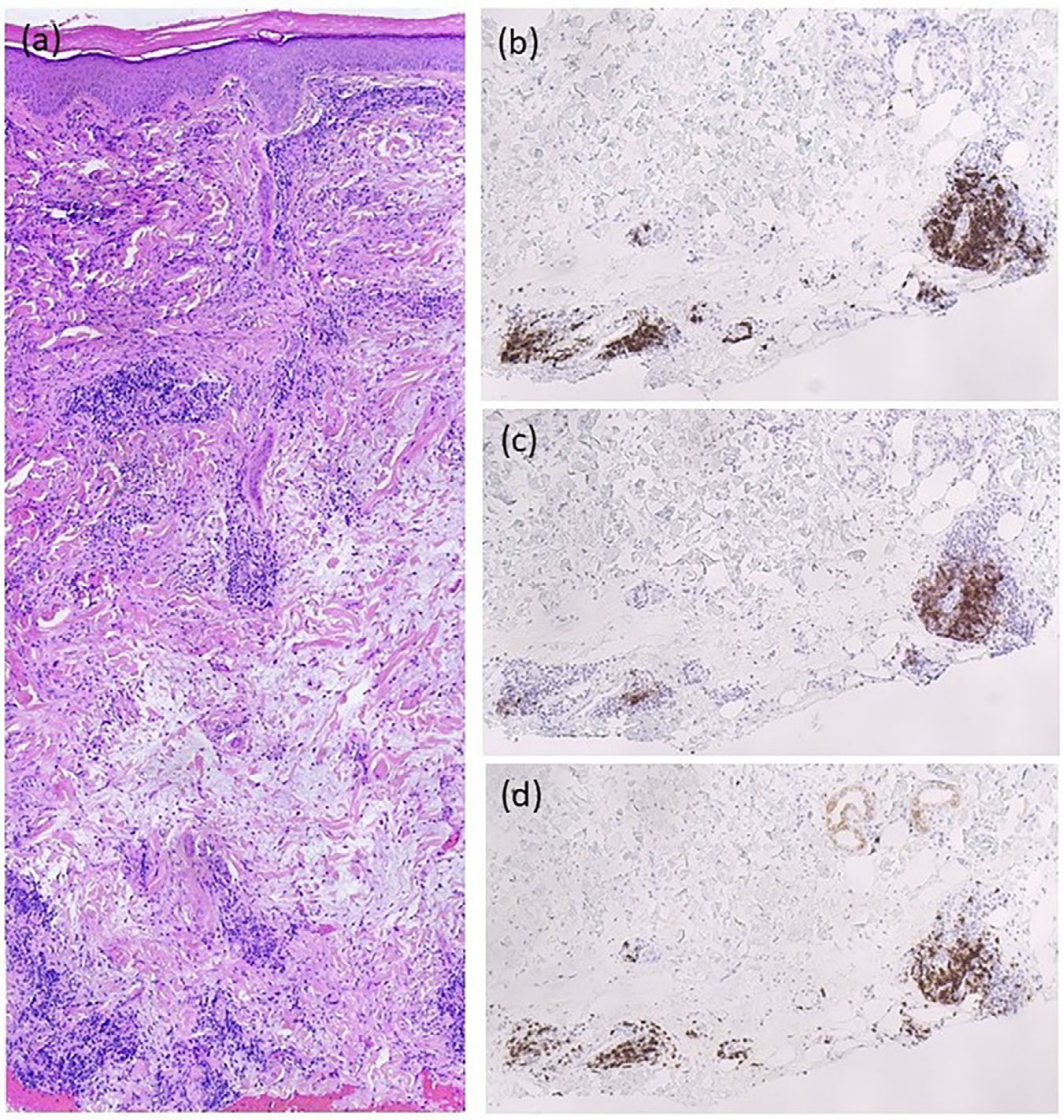
Histopathological examination of the skin biopsy revealed superficial and deeper perivascular lymphocytic infiltration, without epidermal involvement (hematoxylin‐eosin staining, original magnification × 100 (a)). Additionally, immunohistochemically, there was a marked expression of (b) CD23, (c) CD20, and (d) CD5 (original magnification × 100).

In light of these findings, the diagnosis of LC in the context of CLL with accompanying ACA was established. As treatment for chronic Lyme disease with doxycycline had already been planned and there was no current indication for CLL‐specific therapy, oral doxycycline treatment was initiated. This resulted not only in a marked fading of the livid plaques (ACA lesions) on the forearms and hands, but also in a reduction of the subcutaneous nodules. For this reason, the doxycycline treatment was continued at a lower dose (100 mg once daily) for 3 months, leading to complete healing of all skin changes (Figure 1c,d). The patient has now been under oncological follow‐up for 2 years and is still free of skin manifestations. During this period, no specific therapy for CLL (watch‐and‐wait strategy) has been conducted, which confirms the successful treatment of LC with purely antibiotic monotherapy.

LC most commonly occurs in acute myeloid leukemia (AML) and is less frequently observed in patients with CLL. Its presence often indicates an advanced or particularly aggressive course of the underlying disease, as is frequently the case in AML. Overall, LC is a sign of extramedullary leukemia activity and indicates an advanced stage of disease.^4^ ACA is a frequently overlooked but serious complication of Lyme disease that requires early treatment. An association between Lyme disease and primary cutaneous B‐cell lymphomas, such as primary cutaneous marginal zone lymphoma, has been described in the literature. Similar to the present case, patients with *B. burgdorferi*‐associated primary cutaneous marginal zone lymphoma showed clinical resolution without recurrence after 3 weeks of oral tetracycline therapy.^5^, ^6^ In addition to Borrelia burgdorferi, other infections such as syphilis and herpesviruses have been described as triggers of similar skin manifestations. This underscores the importance of comprehensive infectious workup when LC is suspected.^1^, ^2^, ^3^ The clinical presentation of LC in the context of CLL is broad, ranging from nonspecific skin changes to aggressive, neoplastic skin infiltrates.^7^ Pseudolymphomatous infiltrates, typically presenting as dense B‐ and T‐cell infiltrates that can mimic hematologic malignancies, should be differentiated from this condition.^8^ The mechanisms underlying this association are not yet fully understood. In addition to inflammatory disruption of the blood–skin barrier, chronic antigenic stimulation of B‐cells by the pathogen – enhanced by Toll‐like receptor (TLR) activation, particularly via TLR9 – has been proposed. This activation leads to an increased clonal expansion of B cells.^9^ In fact, skin infiltrates of CLL can also be triggered by topical imiquimod therapy.^10^ Furthermore, Borrelia lipoproteins such as OspA and OspC can act as activators of TLR2, thereby releasing pro‐inflammatory cytokines such as TNF‐α, IL‐6, and IL‐1β. This can modify the tumor microenvironment accordingly.^11^ Drawing parallels to *Helicobacter pylori*‐associated MALT lymphomas, antibiotic therapy for eradicating the underlying infection could, as shown in our case, be a promising treatment option.^12^

Our case report illustrates the rare phenomenon of LC in the context of CLL and a *B. burgdorferi* infection. Early diagnosis and appropriate treatment of the underlying infection have a significant impact on the clinical course of the malignant disease.

## CONFLICT OF INTEREST STATEMENT

None.
